# 3D Adipose Tissue Culture Links the Organotypic Microenvironment to Improved Adipogenesis

**DOI:** 10.1002/advs.202100106

**Published:** 2021-06-24

**Authors:** Joanne X. Shen, Morgane Couchet, Jérémy Dufau, Thais de Castro Barbosa, Maximilian H. Ulbrich, Martin Helmstädter, Aurino M. Kemas, Reza Zandi Shafagh, Marie‐Adeline Marques, Jacob B. Hansen, Niklas Mejhert, Dominique Langin, Mikael Rydén, Volker M. Lauschke

**Affiliations:** ^1^ Department of Physiology and Pharmacology Karolinska Institutet Stockholm 171 77 Sweden; ^2^ Department of Medicine Huddinge Karolinska Institutet Karolinska University Hospital Stockholm 141 86 Sweden; ^3^ Inserm Institute of Metabolic and Cardiovascular Diseases (I2MC) UMR1297 Toulouse 31432 France; ^4^ Université de Toulouse Université Paul Sabatier Faculté de Médecine, I2MC UMR1297 Toulouse 31432 France; ^5^ Renal Division Department of Medicine University Hospital Freiburg and Faculty of Medicine University of Freiburg Freiburg 79106 Germany; ^6^ BIOSS Centre for Biological Signalling Studies University of Freiburg Freiburg 79104 Germany; ^7^ Division of Micro‐ and Nanosystems KTH Royal Institute of Technology Stockholm 100 44 Sweden; ^8^ Department of Biology University of Copenhagen Copenhagen 2100 Denmark; ^9^ Toulouse University Hospitals Department of Biochemistry Toulouse 31079 France

**Keywords:** adipose stem cells, fat cells, lipidomics, organotypic cell culture, preadipocytes, stromal vascular fraction, transcriptomics

## Abstract

Obesity and type 2 diabetes are strongly associated with adipose tissue dysfunction and impaired adipogenesis. Understanding the molecular underpinnings that control adipogenesis is thus of fundamental importance for the development of novel therapeutics against metabolic disorders. However, translational approaches are hampered as current models do not accurately recapitulate adipogenesis. Here, a scaffold‐free versatile 3D adipocyte culture platform with chemically defined conditions is presented in which primary human preadipocytes accurately recapitulate adipogenesis. Following differentiation, multi‐omics profiling and functional tests demonstrate that 3D adipocyte cultures feature mature molecular and cellular phenotypes similar to freshly isolated mature adipocytes. Spheroids exhibit physiologically relevant gene expression signatures with 4704 differentially expressed genes compared to conventional 2D cultures (false discovery rate < 0.05), including the concerted expression of factors shaping the adipogenic niche. Furthermore, lipid profiles of >1000 lipid species closely resemble patterns of the corresponding isogenic mature adipocytes in vivo (*R* ^2^ = 0.97). Integration of multi‐omics signatures with analyses of the activity profiles of 503 transcription factors using global promoter motif inference reveals a complex signaling network, involving YAP, Hedgehog, and TGF*β* signaling, that links the organotypic microenvironment in 3D culture to the activation and reinforcement of PPAR*γ* and CEBP activity resulting in improved adipogenesis.

## Graphical abstract

A scaffold‐free high‐throughput compatible 3D human adipocyte culture platform in chemically defined media is presented. The model is long‐term stable and closely resembles human adipocytes in vivo on transcriptomic, lipidomic, and functional level. Furthermore, transcription factor activity analysis reveals a complex signaling network that links the organotypic microenvironment to improved adipogenesis.

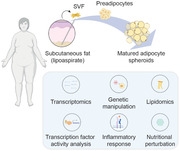

## 1 Introduction

In recent years it has become clear that white adipose tissue (WAT) has a multitude of functions in human health and disease. Adipocytes are critical for lipid storage and release, and WAT constitutes the largest endocrine organ.^[^ ^1^ ^]^ Besides adipokines and cytokines, adipocytes secrete extracellular vesicles with important effector functions across a variety of target tissues.^[^ ^2^ ^]^ The importance of healthy adipose tissue is evident from syndromes and diseases linked to its dysregulation but also from epidemiological studies demonstrating that the prevalence of obesity closely parallels the increasing incidence of cardiovascular diseases, type 2 diabetes mellitus, and non‐alcoholic fatty liver disease.^[^ ^3^, ^4^ ^]^ In metabolically unhealthy obesity, the storage capacity of WAT is exceeded and fat “spills over” into ectopic depots, such as into liver, skeletal muscle, and heart, where it can entail insulin resistance, inflammation, and tissue injury.

The lack of adequate WAT models that allow the stable culture of fat cells, with physiologically relevant phenotypes ex vivo, hampers further insights into adipose biology and the molecular networks underlying adipocyte differentiation, primarily because no model system can fully recapitulate in vivo adipogenesis. The most commonly used adipocyte models are based on the in vitro differentiation of progenitor cells, such as the murine cell line 3T3‐L1, or the human PAZ6^[^ ^5^ ^]^ or Chub‐S7 cell lines.^[^ ^6^ ^]^ While these and other immortalized cell lines are widely available, can be expanded indefinitely, and are easy to use, they differ significantly from their in vivo counterparts in both molecular and cellular phenotypes.^[^ ^7^ ^]^ Primary adipocyte progenitors can be obtained from the stromal vascular fraction (SVF) of human WAT. These cells can be efficiently differentiated into cells with features of mature adipocytes and have been successfully used for mechanistic studies of adipocyte biology.^[^ ^8^, ^9^ ^]^ Furthermore, immortalized human adipose‐derived stem cells (hASCs) constitute additional alternatives that can retain depot‐specific features^[^ ^10^ ^]^ and the ability to acquire beige or brown adipocyte phenotypes.^[^ ^11^ ^]^ However, in conventional 2D monolayer cultures, none of these models exhibit cellular phenotypes, such as large unilocular lipid droplets or physiological gene expression profiles similar to those of mature adipocytes in vivo. As an alternative to differentiation based methods, membrane mature adipocyte aggregate cultures (MAAC) allow for the culture of mature adipocytes under permeable membranes.^[^ ^12^ ^]^ While these cultures exhibit mature adipocyte gene signatures for several days, this method requires freshly prepared material and molecular phenotypes deteriorate considerably after 1 week in culture.

To more closely recapitulate the in vivo microenvironment, cells can be cultured in 3D, allowing for organotypic cell‐cell and cell‐matrix interactions. Compared to 2D cultures, both 3T3‐L1 and hASCs differentiated in 3D acquire phenotypes closer to mature adipocytes.^[^ ^13^, ^14^, ^15^ ^]^ Yet, their resemblance to in vivo counterparts remains distant, with cells displaying considerable multilocularity, low adipokine secretion, and limited lipid accumulation.^[^ ^16^, ^17^, ^18^, ^19^, ^20^ ^]^ While the scaffold‐free culture of murine SVF‐derived adipocyte spheroids using hanging‐drops has shown a modest increase of key adipocyte markers compared to conventional 2D culture for mouse SVF,^[^ ^21^ ^]^ human SVF adipocyte spheroids have so far only been formed in matrigel with serum‐containing media and lack comprehensive phenotypic characterization.^[^ ^22^ ^]^

Here, we addressed the lack of appropriate models and tools for the study of white adipocyte biology. Specifically, we developed a 3D culture platform optimized for scaffold‐free spheroid culture of human and murine adipocyte cell models from various sources. We comprehensively benchmarked the model by integrating systems biology multi‐omics profiling with a battery of functional assessments and demonstrate that molecular and cellular phenotypes closely resemble isogenic freshly isolated mature adipocytes and remained stable for at least 6 weeks in culture whereas conventional 2D cultures, tissue explants, and MAAC deteriorated rapidly. Global activity profiling of >500 transcription factors based on promoter motif activities revealed a critical role of the organotypic microenvironment in 3D culture, comprising fine‐tuned expression of fibronectin, collagens, laminins, and extracellular remodelers, for the activation and reinforcement of key pro‐adipogenic factors, including PPAR*γ* and CEBP.

## 2 Experimental Section

### 2.1 Primary Human White Adipocyte Progenitor Cells

Primary human adipocyte progenitors from the SVF obtained from abdominal subcutaneous WAT (scWAT) of 16 donors undergoing cosmetic surgery (**Table** **1**) were isolated as previously described.^[^ ^23^ ^]^ In brief, the SVF containing adipocyte progenitors were isolated by type 1 collagenase digestion, cells were resuspended in fetal bovine serum (FBS; Hyclone, SV30160.03), and then either cryopreserved for later use or seeded as 2D monolayer or 3D spheroid cultures. As previously reported, SVF consisted of adipocyte progenitors (CD45‐/CD34+/CD31‐; 70–75%), macrophages (CD45+/CD14+; 10%) and leukocytes (CD45+/CD14‐; 7%), while endothelial cells (CD45‐/CD34+) constitute less than 2%.^[^ ^24^ ^]^ Notably, the composition and stoichiometry of SVF were not affected by cryopreservation. All donors gave informed written consent to donate the tissue and the study was approved by the regional board of ethics (permit numbers 2009/764‐32, 2009/1881‐31/1, and 2010/0009‐32). All donors were healthy and had no cardiometabolic or malignant disease.

**Table 1:** Donor information. BMI = body mass index

Donor	Sex	Age	BMI	Surgery type	Fresh/cryopreserved
1	F	46	23.3	Liposuction, cosmetic	Fresh
2	F	56	24.2	Cosmetic	Cryopreserved
3	F	52	24.5	Cosmetic	Fresh
4	F	41	20.2	Cosmetic	Fresh
5	M	48	26.2	Cosmetic	Fresh
6	F	41	30.9	Liposuction, cosmetic	Fresh
7	F	33	22.2	Liposuction, cosmetic	Fresh
8	F	37	25.6	Cosmetic	Cryopreserved
9	F	46	26.4	Liposuction, cosmetic	Fresh
10	F	35	24.0	Cosmetic	Cryopreserved
11	F	45	24.6	Cosmetic	Cryopreserved
12	F	28	20.4	Cosmetic	Cryopreserved
13	F	34	23.7	Cosmetic	Cryopreserved
14	F	43	28.3	Cosmetic	Cryopreserved
15	F	54	21.0	Cosmetic	Cryopreserved
16	F	40	27.3	Liposuction, cosmetic	Cryopreserved

### 2.2 Primary Mouse White Adipocyte Progenitor Cells

Mice were housed and manipulated according to Inserm guidelines and European Directive 2010/63/UE in the local animal care facilities (agreements A 31 555 008, A 31 555 04, and C 31 555 10). Protocols were approved by the French Ministry of Research after review by local ethical committee (CEEA122). Primary adipocyte progenitors from the SVF of wild type and Lipe knockout mice were isolated as previously described.^[^ ^25^ ^]^ In brief, freshly isolated WAT was digested with type II collagenase (Sigma) and SVF was seeded in DMEM containing 10% FBS and 0.5% gentamycin.

### 2.3 Spheroid Culture

Fresh or cryopreserved human primary SVF cells were seeded in culture flasks containing DMEM/F‐12 GlutaMAX (10% FBS, 1% penicillin‐streptomycin). After 48 h, the medium was changed and cells were grown to ≈70% confluence. Subsequently, cells were trypsinized, seeded into 96‐well round bottom ultra‐low attachment microplates (Corning) with 5000 cells per well, and centrifuged at 150 g for 2 min. 4 days after spheroid seeding, cultures were changed to serum‐free differentiation medium consisting of maintenance medium (William's E supplemented with 2 mm L‐glutamine, 100 units mL^−1^ penicillin, 100 µg mL^−1^ streptomycin, 10 µg mL^−1^ insulin, 10 µg mL^−1^ transferrin, 6.7 ng mL^−1^ sodium selenite, and 100 nm dexamethasone) with the addition of a differentiation cocktail consisting of 500 µm 3‐isobutyl‐1‐methylxanthine (IBMX), 10 nm hydrocortisone, 2 nm 3,3′,5‐Triiodo‐L‐thyronine, 10 µm rosiglitazone, 33 µm biotin and 17 µm pantothenic acid. Half of the medium was changed after 48 h and subsequently every 3–4 days. After 17 days of differentiation, the medium was changed to serum‐free maintenance medium without differentiation additives. For supplementation with free fatty acids (FFAs), 160 µm oleic acid and 160 µm palmitic acid conjugated to 10% bovine serum albumin (BSA) at a molar ratio of 1:5 for 2 h at 40 °C before were added to the medium as indicated.

Human preadipocytes from WAT immortalized by telomerase reverse transcriptase (TERT‐hWA) were cultured as previously described.^[^ ^11^ ^]^ Spheroids were formed and differentiated as described above, with some modifications of the differentiation medium: 0.5 µg mL^−1^ insulin, 45.5 µg mL^−1^ transferrin, 0.5 ng mL^−1^ sodium selenite, 5 mm IBMX, and 100 nm hydrocortisone.

Mouse preadipocytes were trypsinized when at ≈70% confluence and cells were resuspended at 1 million cells per mL in basic medium. Hanging drops of 20 µL containing 20 000 cells were generated and spheroid formation occurred within 3–4 days. Spheroids were then differentiated in basic medium supplemented with 17 nm insulin, 100 nm dexamethasone, 125 µm IBMX, 1 µm rosiglitazone during 10 days with medium change every 3–4 days. After the differentiation period, basic medium supplemented with only 17 nm insulin was used for maintenance and spheroids were cultured until day 20.

### 2.4 2D Monolayer Culture

Primary and immortalized human white adipocyte progenitor cells were expanded and cultured as above. However, instead of transfer to ultra‐low attachment plates, preadipocytes were differentiated in monolayer culture using the same differentiation cocktail for 17 days (or for 13 days in the case of TERT‐hWA cultures). As cells in 2D culture progressively detach, monolayer cultures could not be maintained for longer.

Primary mouse white adipocyte progenitor cells were expanded and cultured as described for the respective murine 3D cultures. However, instead of trypsinization and spheroid formation, preadipocytes were differentiated in monolayer culture for 20 days using the same differentiation cocktail.

### 2.5 Viability and Functional Assays

Cell viability was measured using CellTiter‐Glo Luminescent Cell Viability Assay reagent (Promega). Intracellular lipids were quantified through spheroid trypsinization and subsequent mechanical dissociation in the presence of AdipoRed Assay reagent (Lonza). Conditioned media were collected 4 days after each medium change for determination of adiponectin concentrations by ELISA (R&D Systems). For lipolysis assays, human differentiated cells (13 days in 2D culture or 17 days in 3D culture) or preadipocytes were incubated in lipolysis medium (DMEM/F12 supplemented with 2% BSA) without (basal) or with 1 or 10 µm isoprenaline for 3 h at 37 °C. Mouse spheroids were incubated in Krebs‐Ringer Buffer supplemented with 2% BSA and 2 mm glucose without (basal) or with 10 µm forskolin. Glycerol content in the media was quantified using Free Glycerol Reagent (Sigma Aldrich) and Amplex UltraRed (Invitrogen), according to the manufacturers’ instructions. Glycerol content was calculated based on a standard curve using Glycerol Standard Solution (Sigma Aldrich). For assessment of insulin sensitivity, insulin in media was phased out over 24 h, followed by 48 h insulin starvation. Spheroids were then stimulated with 100 nm insulin for 10 min before being harvested for protein extraction. For Western blotting, 5 µg of protein per sample were loaded and membranes were probed with antibodies against pAKT (Ser473, #4060, Cell Signaling Technology) and vinculin (ab129002, Abcam). Bands were detected using the SuperSignal West Femto Maximum Sensitivity Substrate (Thermo Scientific). For cytokine stimulation, spheroids were exposed to IL‐1*β* (10 ng mL^−1^ in maintenance medium) for 48 h. Glucose uptake was quantified as previously described.^[^ ^26^ ^]^

### 2.6 qRT‐PCR

Total RNA was isolated using QIAzol Lysis Reagent and RNA was reverse transcribed into cDNA using SuperScript III Reverse Transcriptase (Invitrogen) or High Capacity cDNA Reverse Transcription Kit (Applied Biosystems, Thermo Fisher Scientific) for human and mouse samples, respectively. qPCRs were performed using TaqMan or SYBR Green Assays utilizing the primers and probes provided in Table S1, Supporting Information.

### 2.7 Brightfield and Fluorescence Imaging

Spheroids were fixed in 10% formalin. For haematoxylin and eosin (H&E) imaging, fixed spheroids were paraffin embedded, sectioned, and processed for H&E staining by the Unit for Morphological Phenotype Analysis (FENO), Karolinska Institutet, Stockholm, Sweden. For confocal imaging, fixed spheroids were stained with 2 µm Nile Red for 24 h at room temperature. Stained spheroids or AAV‐GFP‐transduced spheroids (AAV; adeno‐associated virus) were imaged using a Zeiss LSM 880 confocal microscope. TERT‐hWA 2D cultures were fixed in 4% paraformaldehyde (PFA) and stained for 5 min with BODIPY (lipid stain) and Hoechst33342 (nuclei) in the dark.

### 2.8 Spinning Disk Confocal Microscopy

Spheroids were fixed in 4% PFA under gentle agitation for 1 h and washed 3× in PBS. The fixed spheroids were stained for 10 min with BODIPY (lipid stain) and DRAQ5 (nuclei) dyes in the dark, with gentle agitation. Subsequently, spheroids were transferred into a 24‐well glass‐bottom plate, fixed to the bottom of the plate with a drop of 1.2% low melting agarose (BioRad), and partially cleared using the SeeBD protocol.^[^ ^27^ ^]^ Spheroids were imaged with a dual spinning disk confocal microscope. Laser power was adjusted to prevent image saturation. Image deconvolution was performed using the Lucy–Richardson method.

### 2.9 Electron Microscopy

Spheroids were fixed in 10% formalin, 2% glutaraldehyde in DPBS overnight at 4 °C. Transmission electron microscopy and scanning electron microscopy (SEM) were performed as previously described.^[^ ^28^ ^]^

### 2.10 AAV Transduction

AAV infections were performed in adipocyte spheroids using serotypes 1, 2, 5, 6, and 8.ape encoding the CMV:eGFP gene. All serotypes were used at multiplicity of infection (MOI) ranging between 10^4^ and 10^6^ viral genomes/cell. On day 13 of differentiation, spheroids were incubated in 100 µL of differentiation medium containing the indicated MOI of the respective AAV serotype. After 24 h, the spheroids were washed twice in pre‐warmed DPBS to remove excess virus and spheroids were kept in differentiation medium until day 17 when spheroids were harvested for imaging or the evaluation of GFP expression by qPCR.

### 2.11 Lipidomics

Spheroids (30–40 per sample) were washed in DPBS and snap‐frozen at the time of sampling. At the time of analysis, samples were subjected to Lipidomic Mass Spectrometry as previously described.^[^ ^29^ ^]^

### 2.12 RNA‐Seq

Total RNA was isolated from 8–16 spheroids (3D culture) or one confluent well of a 6‐well plate (2D culture), corresponding to 40 000–80 000 cells. RNA sequencing by poly‐A capture was performed for seven donors by the National Genomics Infrastructure facility at Science for Life Laboratory, Stockholm, Sweden, using a minimum of 100 ng RNA input material. The results were integrated with available RNA‐Seq data from additional preadipocytes, 2D differentiated adipocytes, MAAC, fresh adipose tissue, and explant samples.^[^ ^12^ ^]^ Genes with an average number of fragments per kilo base per million mapped reads *>*1 across all samples were analyzed using Qlucore (Lund, Sweden). Differential gene expression analysis was conducted using DESeq2^[^ ^30^ ^]^ and results were corrected for multiple tests using the Benjamini–Hochberg method with false discovery rates (FDRs) ≤ 5%. Significantly enriched pathways were identified based on the KEGG Pathway Database using the WebGestalt tool box.^[^ ^31^ ^]^

### 2.13 Transcription Factor Activity Analyses

The activity profiles of 503 transcription factors were analyzed during adipocyte differentiation in 2D monolayer and 3D spheroid culture using the ISMARA algorithm by inferring motif activities based on a linear model with a Bayesian procedure where a Gaussian prior on motif activities was used to avoid overfitting.^[^ ^32^ ^]^ The parameters of the prior distribution of the Gaussian likelihood model were estimated using cross‐validations in which activities were inferred on a randomly selected subset of 80% of promoters and the parameters corresponding to the optimal fit were validated using the remaining 20% of promoters. Transcription factor binding motifs were validated using human ChIP‐Seq data in JASPAR.^[^ ^33^ ^]^

### 2.14 Statistical Analysis

Results are presented as mean ± SEM unless stated otherwise. For determining statistical significance, unpaired two‐tailed heteroscedastic *t*‐tests were performed using GraphPad Prism (version 9.0.0) with n≥3 samples per group unless indicated otherwise. All data are shown and no outlier removal was performed. For all data, significance was defined as p ≤ 0.05.

## 3 Results

### 3.1 Primary Human ASC‐Derived Adipocytes Acquire Mature Adipocyte Characteristics in 3D Culture

**Figure 1:**
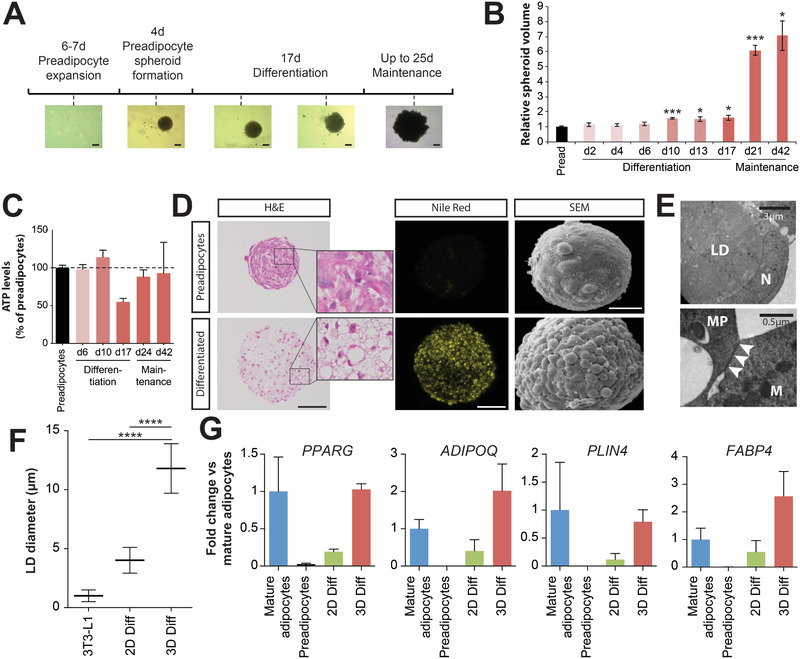
Adipocyte spheroids exhibit long‐term viability and mature adipocyte characteristics. A) Schematic depiction of the spheroid aggregation process. Primary human preadipocytes from SVF are expanded for 6–7 days before the cells are seeded for spheroid formation (<4 days). Cells are differentiated in spheroid conformation for 17 days, after which they are phenotypically stable in maintenance medium without differentiation factors for a further 25 days. Representative brightfield images are shown. All scale bars = 100 µm. B) Spheroid volumes increase significantly starting at day 10 of differentiation. *n* = 6–14 except for day 42 where *n* = 3. * and *** indicates *p* < 0.05 and *p* < 0.001, respectively, of a two‐tailed *t*‐test compared to preadipocyte spheroids. C) Adipocyte spheroids remain viable for at least 42 days of culture after the start of differentiation. *n* = 13–30 except for day 42 where *n* = 3. D) Haemotoxylin and eosin (H&E) staining of preadipocyte and adipocyte spheroids showed an increase in lipid droplets in adipocyte spheroids (left; scale bar = 100 µm). Lipid accumulation is furthermore shown using Nile Red staining (middle; scale bar = 100 µm). Scanning electron microscopy (SEM) visualizes spheroid hypertrophy and increased roughness with looser cellular packing in differentiated spheroids (right; scale bar = 50 µm). E) Transmission electron microscopy images showing the characteristic cellular morphology with a large droplet (LD) that pushes the nucleus (N) against the plasma membrane. Adipocytes form gap junctions (indicated by arrows) and display turnover of mitochondria, termed mitophagy (MP), in which an autolysosomal membrane is enclosing a mitochondrion (M). F) Adipocyte spheroids displayed lipid droplets with diameters that surpass conventional adipocyte culture systems. *n* = 50 for 3T3‐L1 cells, *n* = 38 for 2D culture and *n* = 15 for spheroids. ****p < 0.0001 two‐tailed heteroscedastic *t*‐tests. G) Transcriptional profiling of the key mature adipocyte markers PPARG, ADIPOQ, PLIN4, and FABP4 by qPCR. *n* = 2–4 biological replicates per group. Note that expression levels in cells differentiated in 3D are substantially higher than in 2D cultures and closely resemble expression in mature adipocytes from the same individual. All data are shown as mean ± SEM.

Stromal vascular cells were isolated from human scWAT. Following an initial in vitro expansion of 1–2 cell divisions (6–7 days) in monolayer culture, preadipocytes were trypsinized and seeded onto ultra‐low attachment surfaces in 96‐well plates at 5000 cells per well, where they readily aggregated into spheroids with diameters of 150–200 µm over the course of 4 days (**Figure** **1**A). Following aggregation, cells were differentiated in chemically defined media for 17 days in 3D culture and maintained for up to 25 days in medium without differentiation factors thereafter (42 days in total after the start of differentiation). During differentiation, the adipocyte spheroids increased substantially in volume with significant changes (*p* < 0.05 compared to preadipocyte spheroids) being apparent as early as day 10 of differentiation (Figure 1B). Interestingly, spheroids of some donors further substantially increased in size to diameters up to 800 µm upon change from differentiation to the maintenance medium. While volumes increased, ATP levels as a proxy for the number of viable cells, remained overall stable during the entire culture period (Figure 1C), suggesting that cell hypertrophy rather than hyperplasia caused the increase in spheroid volume. Furthermore, sectioning of tens of spheroids did not show any evidence for the formation of necrotic cores.

Notably, after differentiation, spheroids consisted of loosely packed uni‐ or paucilocular cells, in which the lipid droplet filled almost the entire cell and squeezed the nucleus against the plasma membrane (Figure 1D,E). Furthermore, adipocytes in spheroid conformation exhibited mitophagy (Figure 1E), reminiscent of adipose tissue whitening in vivo.^[^ ^34^ ^]^ Some but not all cells featured unilocular lipid droplets with diameters of 11.8 µm ± 2.1 µm SD, which was substantially larger than droplets in 2D monolayer culture (4 µm ± 1.1 µm SD; *p* < 0.0001) or in conventional cell culture models, such as 3T3‐L1 cells (*∅* = 1 µm ± 0.5 µm; Figure 1F). Droplets in spheroids were furthermore larger than in brown (average *∅* = 3 µm) or beige (average *∅* = 6 µm) adipocytes but remained smaller than in human WAT in vivo (*∅* = 20–150 µm; ref. [35]) or in silk‐scaffolded adipose tissue explant cultures (∅ ≈50 µm; ref. [36]).

We then evaluated the expression of the white adipocyte marker genes PPARG, ADIPOQ, PLIN4, and FABP4 in conventional 2D monolayers and 3D spheroid culture and compared levels to those found in freshly isolated mature adipocytes from the same individuals (Figure 1G). Importantly, expression levels in 3D cultures for all four genes resembled levels found in mature isogenic adipocytes, whereas expression in conventional 2D monolayer cultures was substantially lower. Combined, these results suggest that 3D spheroid culture significantly improves differentiation and results in cells with molecular and cellular features of mature adipocytes.

### 3.2 Adipocyte Spheroids Closely Resemble Mature Adipocytes at the Transcriptomic Level

**Figure 2:**
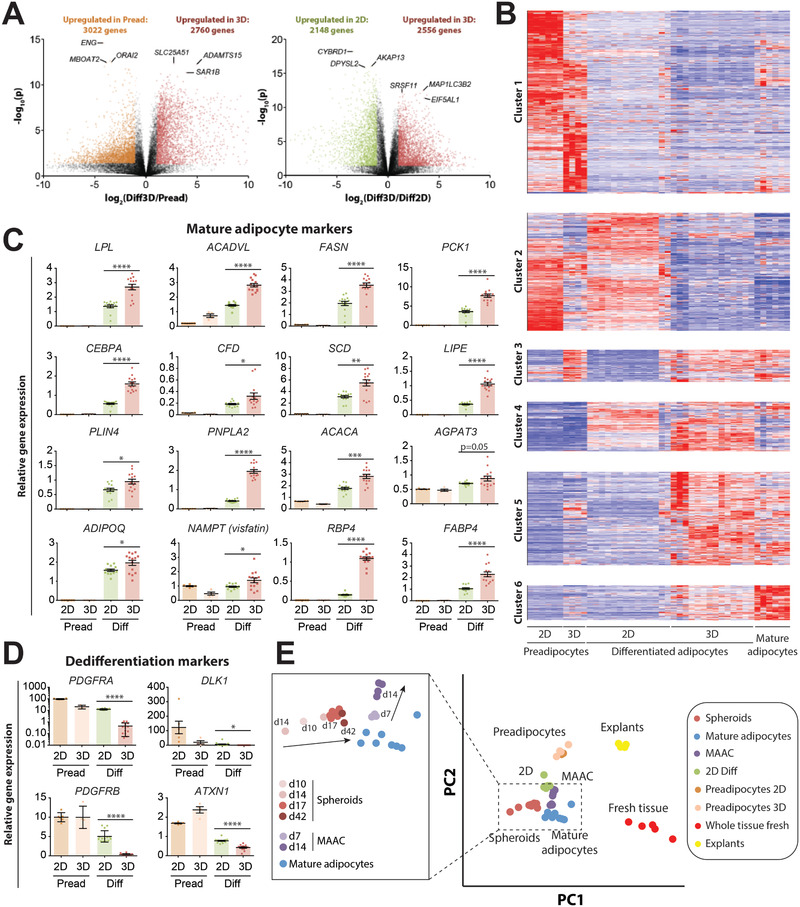
Transcriptomic signatures show that adipocyte spheroids closely resemble mature in vivo adipocytes. A) Volcano plots depict the extent of transcriptomic remodeling during adipocyte differentiation. Differential gene expression is shown for 3D adipocyte spheroids versus preadipocytes (left panel) and 3D spheroids versus 2D adipocytes (right panel). Genes with *p* < 0.05 and fold change > 2 in the respective condition are colored. The top differentially expressed genes are indicated. Note that more than 4500 genes are differentially expressed between cells differentiated in 2D and 3D culture. B) Heatmap representation of hierarchically clustered differentially expressed genes (*F*‐test; q < 0.05) resulted in the formation of six distinct clusters. Compare Table 2 for corresponding pathway analyses. C) Note that expression of mature adipocyte markers was significantly increased in adipocyte spheroids (3D Diff) compared to cells differentiated in 2D (2D Diff), as well as to preadipocytes regardless of culture format. D) Expression of dedifferentiation markers was significantly reduced, corroborating increased maturation in 3D culture. Data are shown as mean ± SEM; *n* = 4–14. *p < 0.05; **p < 0.01; ***p < 0.001; ****p < 0.0001 using two‐tailed heteroscedastic *t*‐tests. E) Principal component analysis of gene expression profiles across different in vitro and ex vivo culture methods. To this end, we integrated our RNA‐Seq data with available data by Harms et al.^[^ ^12^ ^]^ Note that transcriptomic signatures of spheroids closely resemble mature human adipocytes. While membrane mature adipocyte aggregate cultures (MAAC) of mature adipocytes rapidly deteriorate, the molecular phenotypes of adipocyte spheroids resemble mature adipocytes more closely with increasing culture time for up to 42 days (insert).

To investigate the impact of 3D culture on expression patterns in more detail, we comprehensively benchmarked human adipocyte spheroids using RNA‐Seq and compared transcriptomic signatures with preadipocytes, conventional 2D monolayer cultures, and freshly isolated primary mature adipocytes. Overall, compared to preadipocytes, differentiation in 3D spheroids resulted in the up‐ and downregulation of 2760 genes and 3022 genes (*p* < 0.05; fold‐change > 2; **Figure** **2**A), respectively, indicating massive transcriptomic alterations during adipocyte differentiation, as was reported previously in 2D culture.^[^ ^37^ ^]^ Among the most significantly upregulated genes in 3D culture were the mitochondrial NAD^+^ transporter SLC25A51, the metalloprotease ADAMTS15, and the small GTPase SAR1B. Strikingly however, expression signatures differed drastically between cells differentiated in 2D monolayers compared to 3D cultures with a total of 4704 genes being identified as differentially expressed (Figure 2A). SRSF11, MAP1LC3B2, and EIF5AL1 were most significantly upregulated in spheroids, whereas CYBRD1, the mechanotransducer AKAP13 and DPYSL2, encoding a collapsin shown to reduce lipid contents by inhibiting PPAR*γ* and CEBP,^[^ ^38^ ^]^ were most upregulated in 2D culture.

To parse the data in more detail, we performed differential gene expression analyses followed by hierarchical clustering (Figure 2B). In preadipocytes we detected a total of 5817 enriched genes (FDR ≤ 5%; cluster 1) irrespective of culture method, which were significantly associated with focal adhesions (FDR = 0.000 02), actin cytoskeleton (FDR = 0.000 02) and extracellular matrix (ECM) receptor interactions (FDR = 0.003; **Table** **2**). In addition, we identified 3296 genes that were highly expressed in 2D culture of both preadipocytes and differentiated adipocytes but not in 3D spheroids or mature adipocytes (cluster 2) with this cluster being enriched in cell cycle‐associated genes (FDR = 5 × 10^–8^), proteolysis (FDR = 0.000 01) and TGF*β* signaling (FDR = 0.011). Interestingly, 688 genes were induced solely by culturing cells in 3D spheroids as compared to 2D monolayer cultures (cluster 3). These genes were significantly enriched in splicing (FDR = 2.5 × 10^–11^) and RNA transport (FDR = 0.003).

**Table 2:** Differentially regulated pathways in preadipocytes, mature adipocytes, as well as 2D and 3D differentiated adipocytes. Only selected differentially regulated pathways are shown. Cluster designations refer to Figure 2B. FDR = false discovery rate

Cluster	Pathway	Number of genes	Enrichment ratio	*p*‐value	FDR
**Cluster 1** (Specific for preadipocytes)	Glycosaminoglycan biosynthesis	10	2.8	0.001	0.016
	Hippo signaling	12	2.3	0.003	0.029
	ECM receptor interaction	29	2.0	0.0001	0.003
	NF*κ*B signaling	32	1.9	0.0001	0.004
	Focal adhesion	66	1.9	9.9 × 10^–8^	0.000 02
	Regulation of actin cytoskeleton	70	1.8	6.1 × 10^–8^	0.000 02
	JAK/STAT signaling	44	1.5	0.002	0.023
	MAPK signaling	71	1.3	0.004	0.036
**Cluster 2** (Specific for preadipocytes and 2D cultures)	Cell cycle	44	2.7	1.5 × 10^–10^	5 × 10^–8^
	Hedgehog signaling	15	2.4	0.0007	0.019
	p53 signaling	22	2.3	0.000 09	0.003
	Ubiquitin mediated proteolysis	41	2.3	1.7 × 10^–7^	0.000 01
	TGF*β* signaling	23	2.1	0.0004	0.011
**Cluster 3** (Specific for 3D cultures)	Spliceosome	22	7.5	7.7 × 10^–14^	2.5 × 10^–11^
	RNA transport	14	3.8	0.000 02	0.003
**Cluster 4** (Specific for differentiated cells)	2‐oxocarboxylic acid metabolism	6	6.8	0.0002	0.004
	Fatty acid biosynthesis	4	6.2	0.003	0.038
	Propanoate metabolism	9	5.7	0.000 02	0.0008
	Branched‐chain amino acid degradation	13	5.5	3.3 × 10^–7^	0.000 03
	Fatty acid metabolism	13	5.5	3.3 × 10^–7^	0.000 03
	PPAR signaling	11	3.0	0.0009	0.019
	Steroid biosynthesis	5	5.3	0.002	0.027
**Cluster 5** (Specific for differentiated spheroids)	TCA cycle	19	6.8	4.2 × 10^–13^	1.7 × 10^–11^
	Oxidative phosphorylation	61	4.9	<1 × 10^–15^	<1 × 10^–15^
	Pyruvate metabolism	16	4.4	1.3 × 10^–7^	0.000 004
	Fatty acid elongation	10	3.6	0.0002	0.004
	PPAR signaling	15	2.2	0.003	0.042
**Cluster 6** (Specific for mature adipocytes)	Lipolysis	9	5.2	0.000 04	0.014
	Mitophagy	8	3.9	0.0009	0.055
	FoxO signaling	13	3.1	0.0003	0.041

Upon differentiation, pathways involved in fatty acid metabolism (FDR = 0.000 03), as well as branched‐chain amino acid degradation (FDR = 0.000 03) were significantly upregulated in both 2D and 3D culture (cluster 4). Furthermore, genes associated with TCA cycle (FDR = 1.7 × 10^–11^) and fatty acid elongation (FDR = 0.004) were upregulated in differentiated cells exclusively in 3D culture and mature adipocytes but not in 2D culture (cluster 5). A subset of genes associated with PPAR signaling was activated in both 2D and 3D culture (cluster 4; *n* = 11; FDR = 0.019), whereas others were found exclusively in 3D culture (cluster 5; *n* = 15; FDR = 0.042). In addition to bona fide markers, such as PPARG and ADIPOQ (compare Figure 1G), genes with significantly elevated expression in differentiated cells in 3D culture included lipases (lipoprotein lipase (LPL), PNPLA2, and LIPE), fatty acid synthase (FASN), various enzymes involved in fatty acid handling and metabolism (FABP4, ACACA, SCD, PCK1, ACADVL, and AGPAT3), adipokines (ADIPOQ, NAMPT, RBP4, and CFD), perilipins (PLIN4) and transcription factors required for terminal adipocyte differentiation (CEBPA; Figure 2C). Conversely, expression of preadipocyte markers, PDGFRA, PDGFRB, DLK1, and ATXN1 was significantly reduced in spheroids (Figure 2D). While markers of endothelial cells (CD31, CD105, and CD144; ref. [39]) and adipose tissue macrophages (CD45, CD11b, CD11c, ARG1, CD301, CD206; ref. [40]) were expressed during preadipocyte aggregation at levels similar to fresh tissue, expression was lost upon adipocyte differentiation in both 2D and 3D culture (Figure S1, Supporting Information).

Overall, 3D spheroids from preadipocyte derived adipocytes resembled mature adipocytes much more closely than conventional 2D cultures and were comparable in molecular phenotype to MAACs after 7 days in culture (Figure 2E). Importantly however, while MAAC expression patterns deteriorated thereafter, the expression signatures of spheroid cultures resembled mature adipocytes more and more closely with prolonged culture time (Figure 2D, inset). These results thus demonstrate that the presented 3D culture method substantially enhances terminal adipogenesis and improves the long‐term maintenance of physiologically relevant adipocyte phenotypes compared to conventional paradigms.

### 3.3 The Cellular Microenvironment in Spheroids Closely Resembles Adipose Tissue In Vivo

We speculated that the molecular differences between 2D and 3D culture might, at least in part, be caused by differences in the cellular microenvironment, as the abnormal accumulation of ECM components and remodelers is associated with adipocyte dysfunction.^[^ ^41^, ^42^ ^]^ Levels of fibronectin (encoded by FN1), one of the most abundant structural components in the adipose ECM and a well‐established inhibitor of adipocyte differentiation,^[^ ^43^ ^]^ were strongly elevated in preadipocytes (288‐fold increase; *p* = 0.008 compared to mature adipocytes) and 2D cultures (33‐fold increase; *p* = 3 × 10^–6^ compared to mature adipocytes), whereas FN1 expression was not significantly different between 3D spheroids and mature adipocytes (*p* = 0.44; Figure S2A, Supporting Information). The collagen microarchitecture modulates tissue stiffness, impacts adipose tissue gene expression and cause adipocyte dysfunction.^[^ ^44^ ^]^ In 3D culture, we observed an overall decrease in collagens and a reorganization of its isoforms from collagen I, III, and VI toward collagen IV (Figure S2B, Supporting Information), which aligns well with previous longitudinal proteomic studies that identified these alterations to underlie lipid droplet accumulation and adipogenesis.^[^ ^45^ ^]^ Similarly, expression of members of the laminin family was more similar between adipocyte spheroids and mature adipocytes compared to 2D cultures (Figure S2C, Supporting Information). In addition to these structural ECM components, 3D spheroids showed physiological expression patterns of matrix metalloproteases and metallopeptidase inhibitors of the TIMP family (Figure S2D,E, Supporting Information). Combined, these findings thus demonstrate that 3D spheroids, in contrast to conventional 2D cultures, reorganize their extracellular microenvironment to closely mimic the organotypic niche in adipose tissue in vivo.

### 3.4 Global Activity Analysis of Transcriptional Motifs Reveals the Regulatory Network Underlying Human Adipocyte Terminal Differentiation

**Figure 3:**
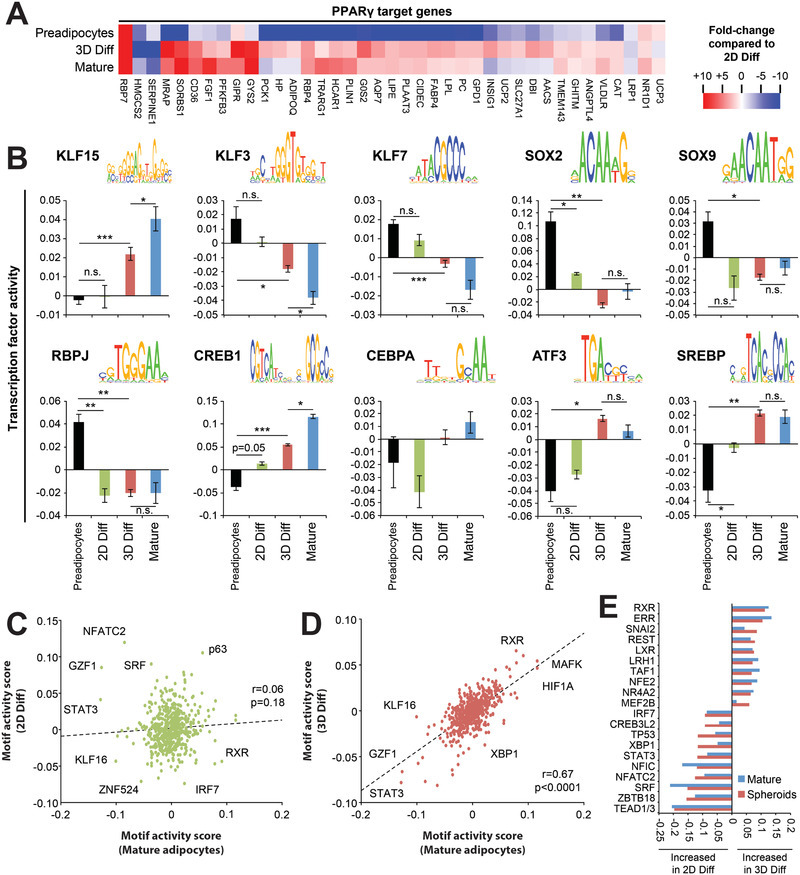
Global activity analysis of transcriptional regulatory motifs demonstrates high concordance between mature human adipocytes and 3D spheroids. A) Heatmap representation of the expression of experimentally validated PPAR*γ* target genes. Expression is shown as normalized to conventional 2D monolayer culture. B) Activity of candidate transcription factors with known importance in adipogenesis. Note that activity signatures in 3D spheroids (3D Diff) resemble mature adipocytes much more closely than 2D cultures (2D Diff) from the same donors. Sequence logos show the binding motif of the respective transcription factor. Data are shown as mean ± SEM; *n* = 4–10 replicates per condition. *, ** and *** indicate *p* < 0.05, *p* < 0.01 and *p* < 0.001, using two‐tailed heteroscedastic *t*‐tests, respectively. n.s. = not significant. Scatter plots showing the correlation between motif activities in mature human adipocytes compared to C) 2D adipocyte cultures or D) 3D adipocyte spheroids. E) The top 10 transcription factors with increased and decreased activities in 2D compared to 3D culture are shown. Activities in mature adipocytes (blue bars) are shown for reference.

To explore the drivers behind the observed transcriptomic differences, we analyzed transcription factor activity. Due to the essential role of PPAR*γ* in adipogenesis, we first focused on the expression of all experimentally validated direct PPAR*γ* target genes (*n* = 44).^[^ ^46^ ^]^ After exclusion of noncoding genes and genes not expressed in human adipocytes, 39 genes (89%) were analyzed. Notably, expression of 27 of these 39 genes was increased at least twofold in 3D spheroids compared to 2D culture (**Figure** **3**A). Among the PPAR*γ* target genes with the highest upregulation were the adipogenic genes GIPR (47‐fold),^[^ ^47^ ^]^ RBP7 (31‐fold),^[^ ^48^ ^]^ G0S2 (sixfold),^[^ ^49^ ^]^ and the fatty acid transporter CD36 (sixfold).^[^ ^50^ ^]^ In contrast, only HMGCS2 (4‐ to 12‐fold), one of the most highly enriched transcripts in human brown adipose tissue,^[^ ^51^ ^]^ and SERPINE1 (six to 16‐fold), an indicator of adipocyte stress^[^ ^52^ ^]^ repressed by thiazolidinedione‐mediated PPAR*γ* activation in vivo,^[^ ^53^ ^]^ were strongly upregulated in 2D culture compared to both mature adipocytes and 3D cultures. These data suggest that both activation and repression of PPAR*γ* targets are amplified in 3D culture.

We then inferred the control of transcriptionally relevant promoter motifs to analyze the activities of other transcription factors with well‐characterized roles in preadipocyte differentiation (Figure 3B). Adipogenesis is controlled by a complex network of transcription factors. Members of the family of Kruppel‐like factors (KLF) have indispensable roles in preadipocyte maintenance and differentiation.^[^ ^54^ ^]^ During differentiation in spheroid culture, the activity of the proadipogenic factor KLF15 was markedly increased, whereas the activity of the antiadipogenic factors KLF3 and KLF7 decreased. Notably, these effects were substantially more pronounced in 3D culture compared to differentiation in conventional 2D monolayers. Similarly, SOX2 and SOX9, which are required for the maintenance of proliferative, PDGFR*α*‐positive adipose precursors,^[^ ^55^, ^56^ ^]^ and the Notch signaling effector RBPJ*κ*, a key factor in adipocyte dedifferentiation,^[^ ^57^ ^]^ were strongly reduced in both 2D and 3D differentiated cells compared to preadipocytes. Pref‐1 (encoded by DLK1; compare Figure 2C) and its direct interaction with fibronectin activates SOX9, jointly acting as a central gatekeeper for adipocyte differentiation,^[^ ^56^, ^58^ ^]^ thus molecularly linking the low‐fibronectin microenvironment in 3D culture to improved adipogenesis. Activities of the important key proadipogenic transcription factors CEBP*α*, CREB, and its target the transcriptional repressor ATF3^[^ ^59^ ^]^ were strongly increased in 3D culture and mature adipocytes, whereas their activation was much less pronounced in 2D culture. Furthermore, we found that the increase of SREBP activity upon differentiation was considerably higher in 3D than in 2D culture.

Next, we performed a comparative global analysis of the activities of 503 transcription factors during differentiation in 2D and 3D culture (Figure 3C,D). Overall, transcription factor activities correlated very poorly between mature adipocytes and preadipocytes differentiated in 2D culture (*r* = 0.06; *p* = 0.18). In contrast, using preadipocytes from the same donors and the identical differentiation protocol, our 3D spheroid model substantially improved the alignment of the regulatory circuitry (*r* = 0.67; *p* < 0.0001). We subsequently investigated which transcription factors might best explain these differences between culture paradigms. The most downregulated transcription factor activities in 2D culture were RXR (Δactivity = −0.11) and its heterodimerization partner LXR (Δactivity = −0.07; Figure 3E), which constitutes a critical promoter of adipogenesis by inducing PPAR*γ*.^[^ ^60^ ^]^ Conversely, 2D cultures lack repression of the activity of transcription factors of the TEAD family (Δactivity = +0.2), which are mediators of Hippo signaling that inhibit adipogenesis by direct binding to the promoters of PPAR*γ*.^[^ ^61^ ^]^ Similarly, 2D cultures featured increased activity of ZBTB18, a repressor of SREBP,^[^ ^62^ ^]^ leading to lower levels of lipid and sterol species compared to spheroids due to attenuated de novo lipogenesis. Combined, these results argue for the presence of a positive feedback loop in which the organotypic microenvironment of the spheroids alleviates inhibition of SREBP, resulting in increased intracellular lipid and sterol levels, which in turn activate LXR signaling, jointly reinforcing PPAR*γ* and CEBP expression and differentiation.

### 3.5 Adipocyte Spheroids Display Relevant Functionality and Are Amenable to AAV Transduction

**Figure 4:**
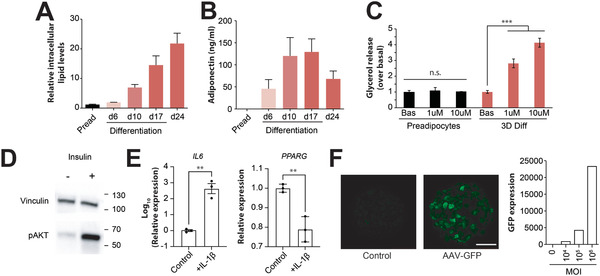
Adipocyte spheroids are functional and amenable to adeno‐associated virus‐mediated gene expression manipulation. A) Intracellular lipids levels continuously increase during differentiation and maintenance. *n* = 12–40. B) Spheroids secrete relevant amounts of adiponectin starting from day 6 of differentiation. *n* = 3. C) Isoprenaline‐induced lipolysis resulted in dose‐dependent increase of glycerol release by adipocyte spheroids but not in preadipocyte spheroids. *n* = 3. D) Western blot images for the insulin signal transducer pAKT shows that adipocyte spheroids remain sensitive to insulin stimulation. E) Stimulation with the inflammatory cytokine IL‐1*β* induced expression of IL6 and downregulated PPARG, demonstrating that phenotypic deterioration during adipose tissue inflammation can be recapitulated ex vivo. *n* = 3. F) Cells can be transduced with adeno‐associated virus (AAV) carrying a GFP expression construct during spheroid formation and GFP fluorescence was detected at day 17 of differentiation. GFP expression was multiplicity of infection (MOI)‐dependent, allowing for quantitative tuning of transgenic expression. All data are shown as mean ± SEM. ** and *** indicates *p* < 0.01 and *p* < 0.001, using two‐tailed heteroscedastic *t*‐tests. n.s. = not significant, respectively.

The functionality of adipocyte spheroids was evaluated using a battery of functional endpoints. Spheroids stored intracellular lipids, and lipid accumulation further increased even after withdrawal of the differentiation cocktail (**Figure** **4**A). Similarly, spheroids produced physiologically relevant levels of the adipokine adiponectin (Figure 4B). Lipolysis constitutes a key function of WAT in which triacylglycerols (TAGs) are hydrolyzed into fatty acids and glycerol. Isoprenaline dose‐dependently stimulated lipolysis‐mediated glycerol release in 3D spheroid culture but not in preadipocytes (Figure 4C). Furthermore, spheroids remained sensitive to insulin stimulation as evidenced by phosphorylation of the downstream signaling protein AKT (Figure 4D). Notably, adipocyte spheroids strongly responded to the inflammatory factor IL‐1*β* by increased expression of IL‐6 (652‐fold; *p* = 0.001) and downregulation of PPARG (0.78‐fold; *p* = 0.006; Figure 4E), as was reported in vivo.^[^ ^63^, ^64^ ^]^

Additionally, we investigated whether adipocyte spheroids may be amenable to AAV transductions to provide a tool kit for gene expression manipulations that would allow for future functional studies. To this end, cells were infected with several different AAV serotypes among which type 6 (AAV6)‐GFP displayed the strongest GFP signal. GFP expression increased dose‐dependently with increasing MOI and GFP signal was detected using fluorescence microscopy, demonstrating that adipocyte spheroids are amenable to viral transductions (Figure 4F).

### 3.6 Adipocyte Spheroids Respond to Nutritional Perturbations

**Figure 5:**
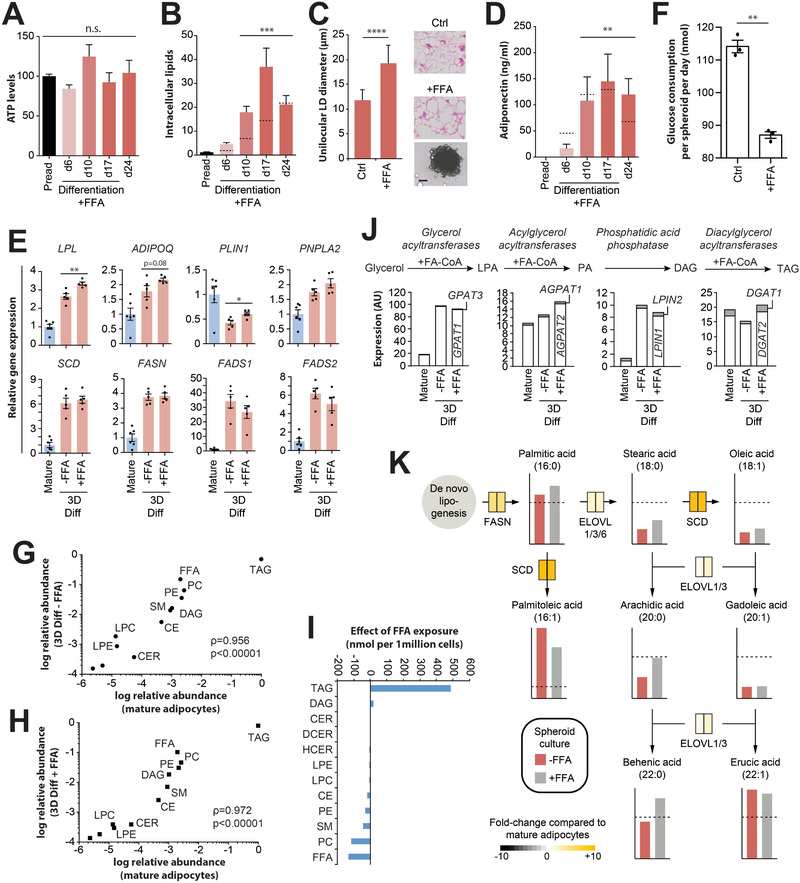
Adipocyte spheroids as a physiologically relevant model to study nutritional perturbations. A) Supplementation of culture medium with free fatty acids (FFAs) did not affect cell viability. *n* = 15–24. B) FFA treatment facilitates lipid loading and adipocyte hypertrophy. *n* = 13–40 spheroids per time point. *p*‐values refer to the comparison of lipid levels for each time point to preadipocytes. Dashed lines indicate intracellular lipid levels in spheroids without FFA supplementation. C) FFA supplementation results in the formation of larger lipid droplets (LD). *n* = 7–11. Scale bar = 100 µm. D) Adiponectin secretion remains unaffected by FFA supplementation. *n* = 3. Dashed lines indicate adiponectin secretion from spheroids without FFA supplementation. *p*‐values refer to the comparison of adiponectin levels for each time point to preadipocytes. E) FFA loading significantly increases expression of LPL and PLIN1, whereas other adipocyte markers (ADIPOQ and PNPLA2) and genes involved in fatty acid handling (SCD, FASN, FADS1, and FADS2) were not affected. *n* = 5–6. *p*‐values refer to the comparison of gene expression levels between spheroids with and without FFA supplementation. F) Glucose consumption decreased upon FFA supplementation, potentially due to insulin resistance caused by lipid overloading.^[^ ^97^ ^]^ *n* = 3. G) Lipidomic analyses revealed that the overall lipid class composition of adipocyte spheroids closely mimics the corresponding signature of isogenic mature adipocytes in vivo (Spearman *ρ* = 0.956). H) Correlations are further improved by FFA supplementation (Spearman *ρ* = 0.972). I) Specifically, FFA exposure increased triacylglycerol (TAG) and diacylglycerol (DAG) levels but decreased intracellular levels of FFA and phosphatidylcholines (PC). J) The molecular machinery involved in TAG synthesis is upregulated compared to mature adipocytes in vivo, facilitating lipid loading, whereas effects of FFA supplementation are overall small. K) Schematic FFA flux analysis in control and FFA treated adipocyte spheroids. Abundances of FFA species are shown as column plots relative to mature adipocytes (dashed lines). Red and grey columns indicate levels of the respective FFA in spheroids without and with FFA supplementation, respectively. Expression of key enzymes is shown in boxes shaded from grey (expression level lower than in mature adipocytes) to yellow (higher). The left and right sections of each box correspond to expression in control and FFA‐supplemented spheroids. All data are shown as mean ± SEM. *, **, *** and **** indicate *p* < 0.05, *p* < 0.01, *p* < 0.001 and *p* < 0.0001, using two‐tailed heteroscedastic *t*‐tests, respectively. n.s. = not significant. PE = phosphatidylethanolamines; SM = sphingomyelins; CER = ceramides; LPC = lysophosphatidylcholines; LPE = lysophosphatidylethanolamines; DCER = dihydroceramides; HCER = hexosylceramides; LCER = lactosylceramides; CE = cholesterol esters.

To evaluate whether spheroids might provide a suitable model for the study of nutritional perturbations in vitro, we studied their response to FFA supplementation. FFAs did not affect spheroid viability but significantly increased the extent of intracellular lipid accumulation and the size of unilocular lipid droplets (**Figure** **5**A–C). Furthermore, FFA loading slightly increased LPL and perilipin (PLIN1) expression, whereas levels of adiponectin and expression of other genes with importance for mature adipocyte function were, if at all, only marginally increased (Figure 5D,E). Interestingly, FFA‐treated adipocytes consumed substantially less glucose compared to control spheroids (Figure 5F), suggesting a further whitening of the adipocyte phenotypes.^[^ ^65^ ^]^

To study the effect of FFAs in more detail, we performed lipidomic analyses of FFA‐treated spheroids and controls. As expected, TAGs were by far the most prevalent lipid class followed by FFAs, phosphatidylcholines (PCs), phosphatidylethanolamines, and diacylglycerols (DAGs) with overall lipid signatures closely resembling their relative abundance in mature adipocytes (Spearman *ρ* = 0.956; Figure 5G). FFA supplementation further increased levels of TAGs, consistent with the increasing size of lipid droplets, and slightly improved correlations with mature adipocyte lipidomic signatures (Spearman *ρ* = 0.972; Figure 5H,I). In contrast, FFA supplementation reduced levels of intracellular FFAs and PCs.

Next, we evaluated whether spheroids could provide tools to study lipid fluxes. To this end, we analyzed fatty acid abundance and associated specific fluxes with expression levels of the respective enzymes. Expression of glycerol acyltransferases and phosphatidic acid phosphatase was increased in 3D culture compared to mature adipocytes, whereas expression of mono‐ and DAG acyltransferases remained unaffected by ex vivo culture (Figure 5J). We compared the intracellular fatty acid profiles in spheroids with and without FFA supplementation to the corresponding patterns in mature adipocytes from the same donors (Figure 5K). Notably, while palmitic acid concentrations were similar, palmitoleic acid (16:1) levels were increased in adipocyte spheroids compared to mature adipocytes, likely at least in part due to overexpression of the responsible enzyme, SCD. Palmitoleate constitutes an FFA of considerable interest because it is secreted and impacts metabolism and insulin resistance in distant tissues.^[^ ^66^ ^]^ Due to high SCD expression, levels of stearic acid (18:0) were lower in spheroids, which mirrors the result from clinical studies showing that levels of stearic acid in WAT correlate inversely with increased fat mass and adipocyte hypertrophy.^[^ ^67^ ^]^ Notably, overall FFA compositions were retained and also substantially less abundant species, such as arachidic acid (20:0), behenic acid (22:0), gadoleic acid (20:1), and erucic acid (22:1) remained detectable in culture at physiological levels. We thus conclude that 3D adipocyte spheroids from human SVF constitute a physiologically relevant organotypic model system that accurately recapitulates the molecular phenotypes of human WAT at the transcriptomic, lipidomic, and functional level.

### 3.7 The Established 3D Spheroid Culture Paradigm Also Improves the Phenotypes of Immortalized Human Adipocyte Models

To test the potential of 3D cultures for improving the adipose phenotype of other cell sources, we used TERT‐hWA, a recently described polyclonal cell model of immortalized human white adipose progenitors.^[^ ^11^ ^]^ Notably, using the spheroid culture method presented above, these cells rapidly aggregated and altered their morphology and lipid droplet characteristics compared to 2D culture (Figure S3A, Supporting Information). Additionally, 3D spheroid cultures from TERT‐hWA displayed an increased concentration‐dependent glycerol release in response to isoprenaline, whereas adiponectin secretion was not significantly affected (Figure S3B,C, Supporting Information). Combined, these results suggest that spheroid culture can also contribute to limited but significant improvements in the functionality of immortalized adipocyte cell lines.

### 3.8 Murine Adipocyte Spheroids Allow for Mechanistic Studies Using Genetic Knock‐Out Models

**Figure 6:**
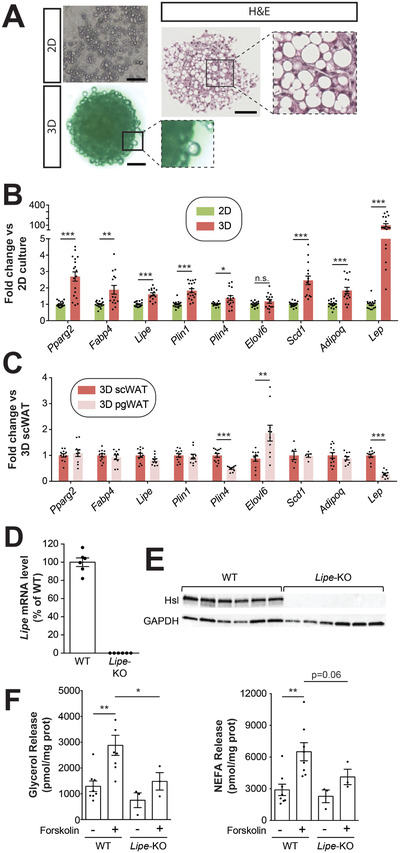
The established spheroid platform improves differentiation of mouse preadipocytes from different depots. A) Brightfield live cell and H&E images of mouse adipocytes differentiated for 20 days in 2D or in 3D spheroid culture. Note the formation of large unilocular cells in 3D culture whereas cells in monolayers remain multilocular. B) Relative RNA expression level of adipocyte markers is shown in conventional 2D and 3D spheroid culture. C) Differences in gene expression are shown between murine adipocyte spheroids differentiated for 20 days from preadipocytes isolated from inguinal subcutaneous (sc) or perigonadal (pg) fat. D) Lipe mRNA and E) hormone‐sensitive lipase (Hsl; encoded by Lipe) protein are not detectable in adipocytes from Lipe‐KO mice. F) Forskolin significantly induced the release of glycerol (left panel) and non‐esterified fatty acids (NEFA; right panel) by adipocyte spheroids from wild type (WT) mice. Notably, lipolysis was significantly reduced in spheroids generated from Lipe‐KO mice. All data are shown as mean ± SEM. *, ** and *** indicate *p* < 0.05, *p* < 0.01 and *p* < 0.001 using two‐tailed heteroscedastic *t*‐tests, respectively.

As transgenic mice are widely used to investigate the role of proteins in adipocyte biology, we utilized the established spheroid model to differentiate mouse white preadipocytes. Notably, in contrast to human adipocyte models, murine stromal vascular cells are known to require serum for differentiation in 2D culture^[^ ^68^ ^]^ and we observed similar serum‐dependency in 3D spheroids. When differentiated in 3D, preadipocytes from inguinal subcutaneous adipose tissue displayed high lipid loading associated with unilocular and paucilocular lipid droplet formation (**Figure** **6**A). In contrast, cells from the same animals differentiated in 2D culture remained multilocular. Consistent with these observations, gene expression profiling of adipogenic markers showed significantly higher expression in spheroids compared to 2D cultures (Figure 6B). Differences were most pronounced for leptin where 3D culture resulted in 99‐fold higher expression in spheroid culture (*p* < 0.001). The higher expression of leptin is in line with the increased fat cell size in 3D culture.

Next, we evaluated whether the spheroid model allowed for studies into differences in expression signatures between WAT depots. While subcutaneous inguinal preadipocytes differentiated well in 2D culture with multilocular lipid droplets being evident in ≈70% of cells, 2D differentiation of perigonadal preadipocytes was poor and only <5% of all cells featured small droplets after 20 days of culture (Figure S4, Supporting Information). In contrast, preadipocytes from both depots differentiated well in 3D culture and most cells featured uni‐ or paucilocular droplets after the same period of differentiation. While the expression of most markers was similar between perigonadal and scWAT, significantly reduced expression in the former was observed for Plin4 (0.5‐fold; *p* = 1.3 × 10^–5^) and Lep (0.27‐fold; *p* = 5.2 × 10^–9^), whereas Elovl6 was significantly increased (2.1‐fold; *p* = 0.01; Figure 6C), suggesting that fat depot‐specific patterns are recapitulated in 3D culture.

Last, we evaluated whether the model could be used for mechanistic studies into adipocyte biology by making use of the extensive repertoire of genetic tools. Specifically, we generated 3D cultures of adipocytes from inguinal adipose tissue of mice homozygous for a null mutation in Lipe, which encodes the lipolytic enzyme hormone‐sensitive lipase (Hsl). We first validated the lack of Lipe expression and absence of Hsl protein in spheroids from Lipe‐KO mice (Figure 6D,E). Next, we evaluated adipocyte lipolytic activity in response to the adenylate cyclase activator forskolin. In 3D adipocyte cultures of WT mice, we observed significant increases in the release of glycerol (*p* = 0.004) and non‐esterified fatty acids (NEFA; *p* = 0.004) upon induction (Figure 6F). However, these responses were blunted by 49% and 37%, respectively in adipose spheroids from Hsl deficient mice. These results align well with previous reports suggesting reduced but not absent lipolytic activities of Lipe‐KO mice.^[^ ^69^ ^]^ We conclude that the established spheroid model provides a versatile, physiologically relevant culture paradigm across cell sources, species, and fat depots that facilitates comparative long‐term studies of adipocyte function and biology.

## 4 Discussion

Physiologically relevant models of WAT are essential for studies of lipogenesis, fat tissue biology, as well as for discovery and development of candidate drugs that target white adipocytes. Previous studies indicated that 3D culture facilitated the formation of unilocular lipid droplets, improved cellular phenotypes compared to conventional monolayer culture, and allowed the long‐term maintenance of SVF‐derived adipocytes for many weeks.^[^ ^70^, ^71^, ^72^, ^73^ ^]^ Furthermore, 3D adipose models have facilitated the study of adipose tissue beiging^[^ ^74^, ^75^ ^]^ and effects of interstitial flow.^[^ ^76^ ^]^ Importantly however, multiple limitations remain, including immature molecular and cellular phenotypes, the need for freshly prepared tissue samples, dependency on serum‐containing media that complicates result interpretation, need for the addition of various scaffolds, as well as the lack of scalability. Here, we developed a scaffold‐free spheroid culture platform in chemically defined conditions for differentiation and maintenance of adipocytes derived from a multitude of different human and murine cell sources. Spheroids showed improved phenotypes compared to conventional 2D and 3D culture paradigms and allow for the facile parallel culture of hundreds of microtissues from a single donor. Furthermore, we established conditions for AAV‐mediated gene expression manipulations, providing a tool kit for future functional tests, for instance by introduction of reporter constructs or gene knock‐downs, in primary human cells. Notably, the maintenance media composition of adipocyte spheroids established here is identical to culture conditions for other primary human tissue models, such as 3D liver cultures,^[^ ^77^, ^78^, ^79^ ^]^ thus opening possibilities for the design of future tissue co‐culture systems to study metabolic disease.

Using primary human adipocyte progenitors from the SVF, we demonstrate with a multitude of orthogonal imaging and molecular biology techniques that adipocytes in spheroids obtain molecular phenotypes closely resembling mature adipocytes from the same donor. Furthermore, lack of induction of SLC2A1, VEGFA, ANGPTL4, and SERPINE1, known markers of hypoxia in human adipocytes,^[^ ^80^ ^]^ suggests normoxic culture conditions, which aligns well with the absence of necrotic core formation as evident from sections. Spheroids could be generated from both fresh and cryopreserved cells, thus allowing to replicate experiments using material from the same donor, and reducing reliability on fresh tissue supply, which is particularly important in times of altered clinical routines and reduced numbers of elective surgeries, as seen for example during the COVID‐19 pandemic. Notably, we did not observe differences in spheroid morphology, viability, or intracellular lipid levels between fresh or cryopreserved adipocytes (*p* > 0.2 for all time points and conditions; *n* = 3 donors per group), suggesting that effects of cryopreservation are, if at all, minor.

Adipocyte sizes in spheroids (20 µm) remain smaller than those of average white adipocytes in vivo. Notably however, in a sex‐, age‐ and BMI‐matched cohort, the fraction of small adipocytes with diameter of 20–40 µm was found to correlate with insulin resistance.^[^ ^81^, ^82^ ^]^ While we show that insulin response is maintained in adipocyte spheroids, the high levels of insulin during adipocyte differentiation might reduce insulin sensitivity compared to cells in vivo, which might entail reduced cell sizes. It will also be interesting to determine in future work whether severe obesity and metabolic disease may impact adipocyte hypertrophy and spheroid functionality. Furthermore, intracellular triglycerides in spheroids are generated mainly by de novo lipogenesis with additional contributions from oleic and palmitic acid uptake upon media supplementation with FFAs. While these constitute the two most abundant fatty acids in human plasma,^[^ ^83^ ^]^ other fatty acids, such as linoleic acid and stearic acid, can also reach considerable quantities. Additionally, as cholesterol constitutes a major determinant of adipocyte size, exposure to low density lipoprotein (LDL) could further increase lipid droplet size, stimulate hypertrophy and increase the fraction of unilocular cells in spheroids.^[^ ^84^ ^]^

Spheroid culture resulted in the increased expression of genes involved in spliceosome and RNA transport, which act as enablers for the massive recalibration of transcriptomic signatures compared to conventional 2D culture. These findings align with previous studies that found these pathways to also be significantly upregulated in other organotypic human 3D culture models compared to their 2D equivalents.^[^ ^85^ ^]^ NAD^+^ constitutes a central cellular energy sensor and reduced NAD^+^ levels in WAT result in inactivation of PPAR*γ*, reduced adiponectin production, and impaired adipocyte hypertrophy.^[^ ^86^, ^87^ ^]^ Notably, the recently identified NAD^+^ transporter SLC25A51^[^ ^88^ ^]^ was among the most strongly upregulated genes in 3D culture, suggesting the establishment of an energetically permissive environment to support adipocyte function. Furthermore, spheroid culture reduced TGF*β* activity, thereby relieving PPAR*γ* repression^[^ ^89^ ^]^ and inducing CEBP*β* transactivation,^[^ ^90^ ^]^ which allows PPAR*γ* and CEBP*β* to jointly orchestrate the expression of its key adipogenic targets, such as SREBP and various KLFs.^[^ ^91^, ^92^ ^]^

**Figure 7:**
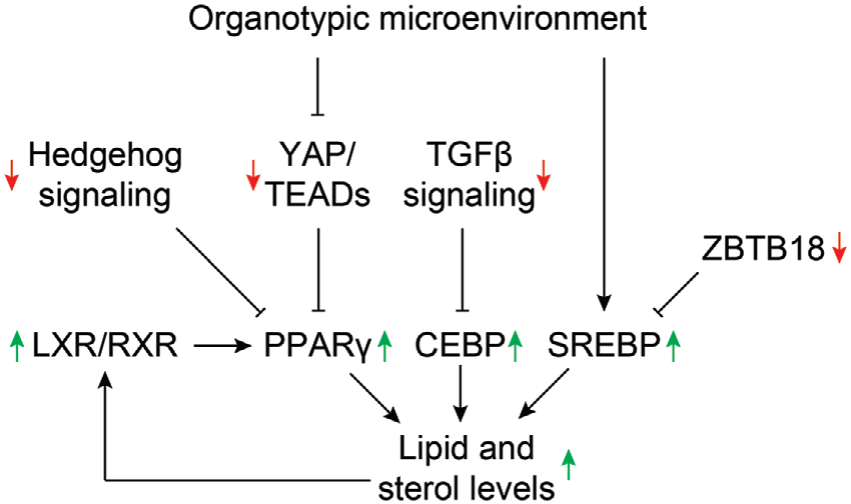
Schematic depiction of the key molecular mediators that link the 3D spheroid microenvironment to phenotypic improvements. Green and red arrows indicate activity of the respective factor or signaling pathway in 3D spheroids compared to 2D culture.

Mechanistically, integrative analyses of transcription factor activity profiles and functional data suggest a model in which the organotypic microenvironment of the spheroid enables activity of SREBP, whose biological function is inhibited by increases of extracellular stiffness,^[^ ^93^ ^]^ and fine‐tunes mechanosensitive TEAD signaling.^[^ ^94^ ^]^ Combined with reduced activity of Hedgehog and TGF*β* signaling, these factors generate a gene regulatory environment permissive for the activation of the key adipogenic factors PPAR*γ* and CEBP*β*, as well as the downstream induction of genes involved in lipid handling. Furthermore, inhibition of ZBTB18, a key repressor of SREBP‐mediated transcriptional activation,^[^ ^62^ ^]^ results in augmented de novo lipogenesis. Intracellular sterol levels, which are considerably higher in 3D adipocytes, in turn activate LXR, and reinforce expression of its targets, including PPAR*γ*, thus providing a positive feedback loop for adipocytic maturation (**Figure** **7**). Combined, our data provide comprehensive molecular evidence for a link between the organotypic microenvironment in 3D culture and terminal adipocyte differentiation during adipogenesis in primary human cells.

We demonstrate that the presented 3D culture paradigm provides a versatile platform to improve the molecular and cellular phenotypes of a variety of adipocyte cell models from diverse origins. However, while cellular phenotypes are highly stable in long‐term culture, the generation of terminally differentiated spheroids takes longer than differentiation using conventional 2D culture protocols (≈30 days in 3D culture including spheroid formation compared to 2 weeks in 2D). We demonstrate functional improvements in adipocyte spheroids differentiated from immortalized preadipocytes compared to conventional 2D culture. Similarly, the setup is compatible with the long‐term culture of murine adipocytes from different fat depots and allows the recapitulation of depot‐specific expression differences in an accessible ex vivo system. This is particularly relevant for the translation of murine studies, which enable the studies of various fat pads.^[^ ^95^, ^96^ ^]^ Additionally, the established spheroid culture provides a platform that combines the accessibility and scalability of an ex vivo model with the extensive genetic tool kit available for mouse research, thus opening new possibilities for functional and translational studies on adipose tissue biology.

In conclusion, we present a versatile scaffold‐free 3D adipocyte culture platform in chemically defined conditions. Using this system, we demonstrate that adipocyte spheroids closely resemble mature adipocytes in vivo and remain phenotypically stable for at least 6 weeks in culture. Using time‐series multidimensional omics profiling, we reveal a complex signaling network, involving YAP, Hedgehog, and TGF*β* signaling, that links the organotypic microenvironment in 3D culture to improved differentiation during human adipogenesis.

## Conflict of Interest

V.M.L. is co‐founder, CEO, and shareholder of HepaPredict AB. In addition, V.M.L. discloses consultancy work for Enginzyme AB. The other authors declare no conflict of interest.

## Author Contributions

J.X.S. developed the 3D spheroid model from human SVF and analyzed the data. M.C. and T.d.C.B. conducted experiments with TERT‐hWA cells and performed lipolysis and AAV transduction assays. J.D. established the mouse adipocyte 3D spheroid model. M.H.U. and M.H. performed electron microscopy. A.M.K. performed glucose consumption quantifications. J.B.H. provided the TERT‐hWA cells. N.M., D.L., and M.R. supervised the research. V.M.L. designed and supervised the research, and analyzed the data. J.X.S. and V.M.L. wrote the manuscript. All authors revised and approved the final version of the manuscript.

## Supporting information

Supporting Information

## Data Availability

Data is shared by the corresponding author upon reasonable request.
