# Supplementary material for: 3D Adipose Tissue Culture Links the Organotypic Microenvironment to Improved Adipogenesis
Source: Adv Sci (Weinh). 2021 Jun 24;8(16):2100106. doi: 10.1002/advs.202100106 (PMC8373086; doi:10.1002/advs.202100106)
Supplement: Supplementary file 1 — Supporting Information [file ADVS-8-2100106-s001.pdf]

© 2021 Wiley-VCH GmbH

ADVANCED SCIENCE
Open Access

Supporting Information

for *Adv. Sci.,* DOI: 10.1002/advs.202100106

3D adipose tissue culture links the organotypic microenvironment to improved adipogenesis

*Joanne X. Shen, Morgane Couchet, Jérémy Dufau, Thais de Castro Barbosa, Maximilian H. Ulbrich, Martin Helmstädter, Aurino M. Kemas, Reza Zandi Shafagh, Marie-Adeline Marques, Jacob B. Hansen, Niklas Mejhert, Dominique Langin, Mikael Rydén, Volker M. Lauschke,**

Supplementary Material

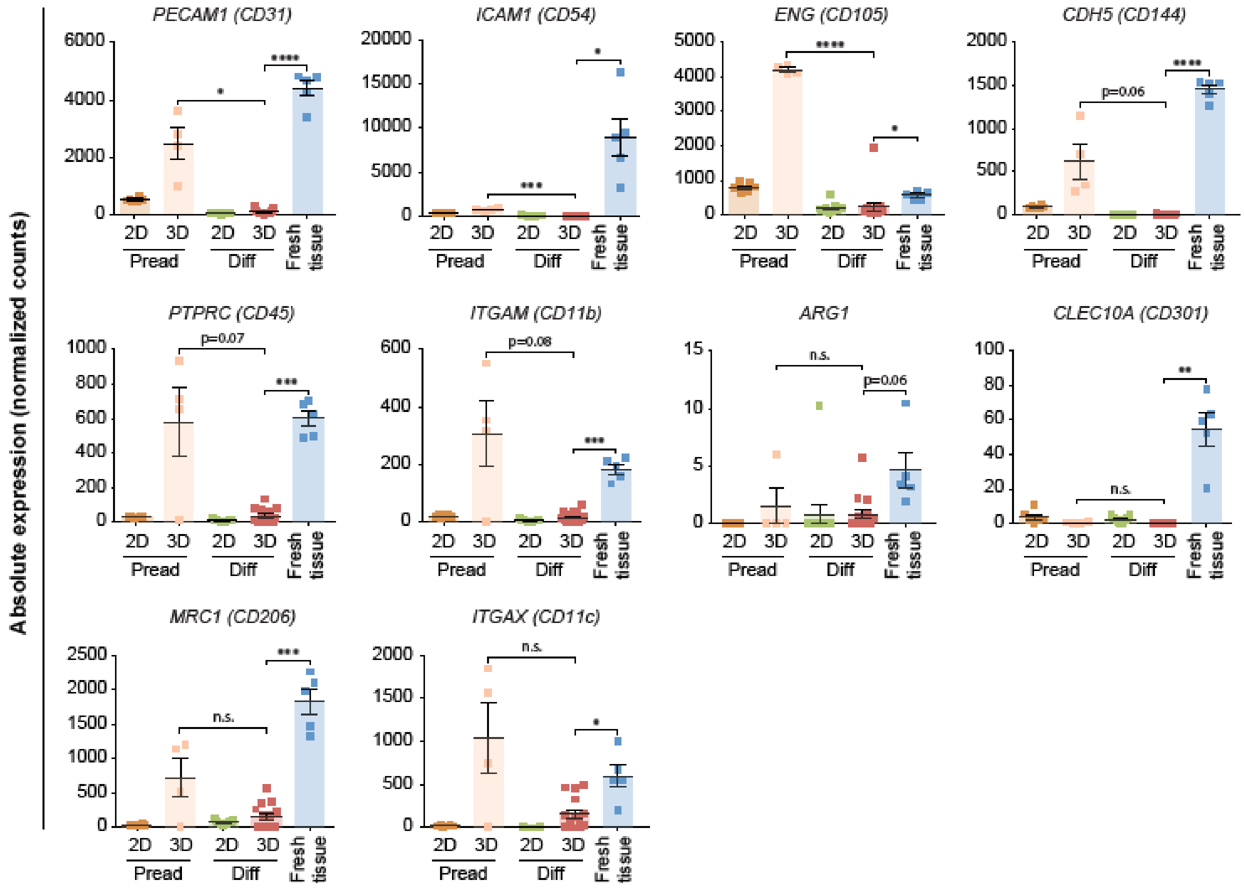

Supplementary Figure 1: Expression of endothelial and macrophage markers. CD31, CD54, CD105 and CD144 constitute markers for adipose endothelial cells, CD45 and CD11b label adipose tissue macrophages, ARG1, CD301 and CD206 label subpopulations of M2 and CD11c of M1 macrophages, respectively. n=6 for 2D preadipocytes, n=4 for 3D preadipocytes, n=15 for adipocyte spheroids (3D Diff), n=13 for 2D adipocytes and n=5 for fresh whole tissue samples. All data are shown as mean ± SEM. *, **, ***, **** indicate $p<0.05$, $p<0.01$, $p<0.001$ and $p<0.0001$ using two-tailed heteroscedastic t-tests, respectively. n.s. = not significant.

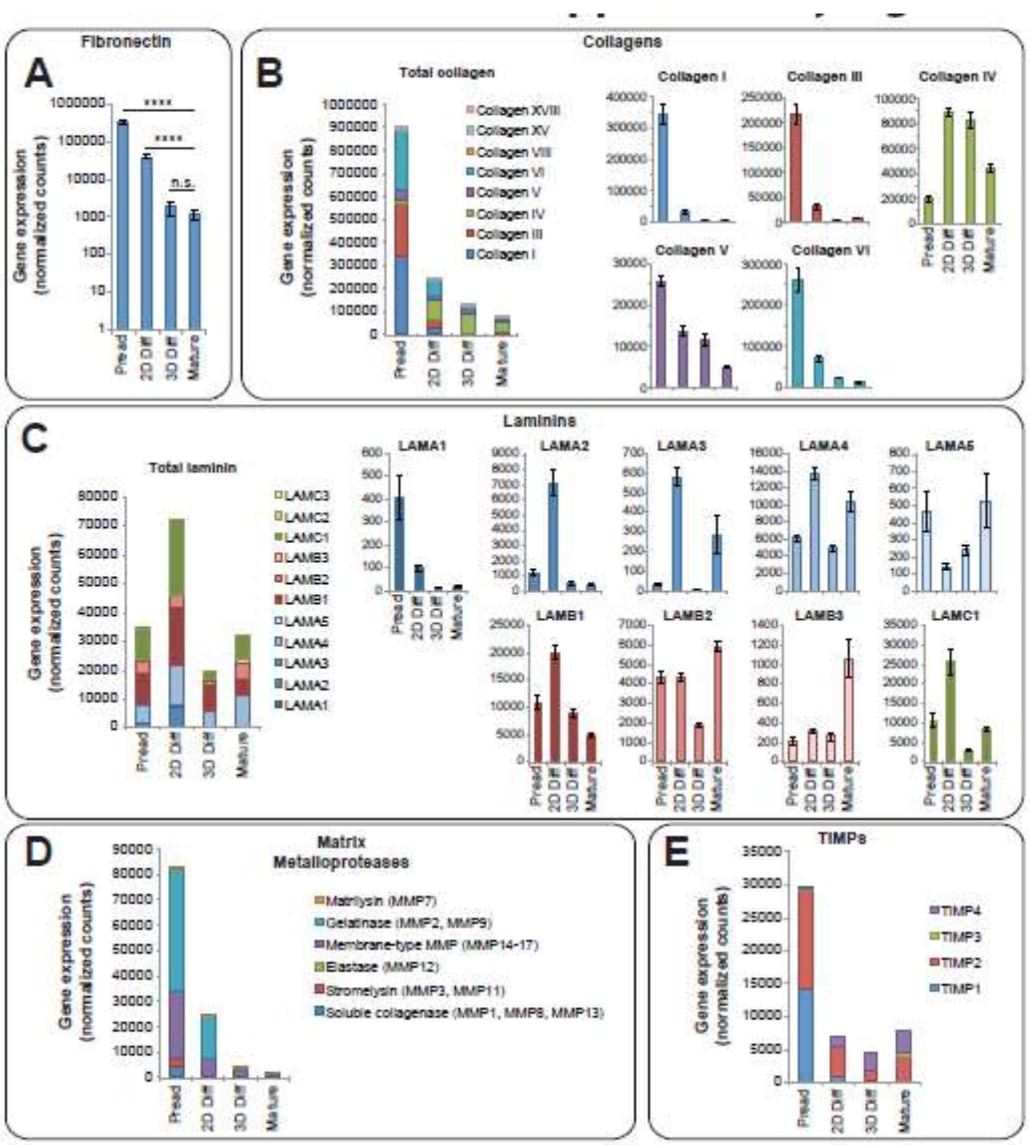

Supplementary Figure 2: Expression profiling of extracellular matrix (ECM) components and remodelers. Expression levels of the structural ECM proteins fibronectin (**A**), collagens (**B**) and laminins (**C**), as well as the major ECM remodeler families of matrix metalloproteases (MMPs; **D**) and tissue inhibitor of metalloproteinases (TIMPs; **E**) are shown in preadipocytes, differentiated adipocytes in 2D and 3D spheroid culture and in mature adipocytes *in vivo*. Expression levels are expressed as normalized gene counts (DeSeq2). n=6 for 2D preadipocytes, n=4 for 3D preadipocytes, n=15 for adipocyte spheroids (3D Diff), n=13 for 2D adipocytes and n=5 for mature adipocytes. All data are shown as mean ± SEM. **** indicate p<0.0001 using two-tailed heteroscedastic t-tests.

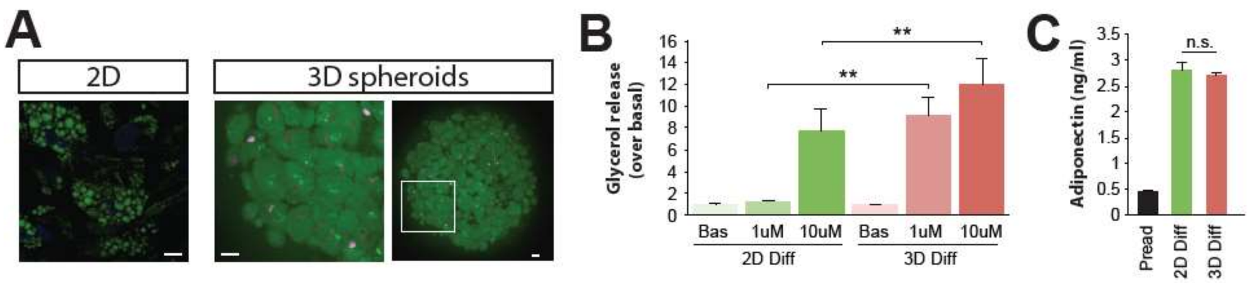

Supplementary Figure 3: Spheroid culture improves phenotypes of immortalized human white adipose tissue cells. A) BODIPY images of TERT-hWA cells in 2D monolayer (left) and 3D spheroid culture (right). **B**) TERT-hWA cells are significantly more susceptible to isoprenaline-induced lipolysis in 3D spheroids compared to 2D monolayer culture. n=5 per condition. **C**) Spheroid culture does not affect TERT-hWA adiponectin secretion compared to 2D monolayer culture. n=4 per condition. Data are shown as mean ± SEM. ** indicate $p<0.01$ using two-tailed heteroscedastic t-tests. n.s. = not significant.

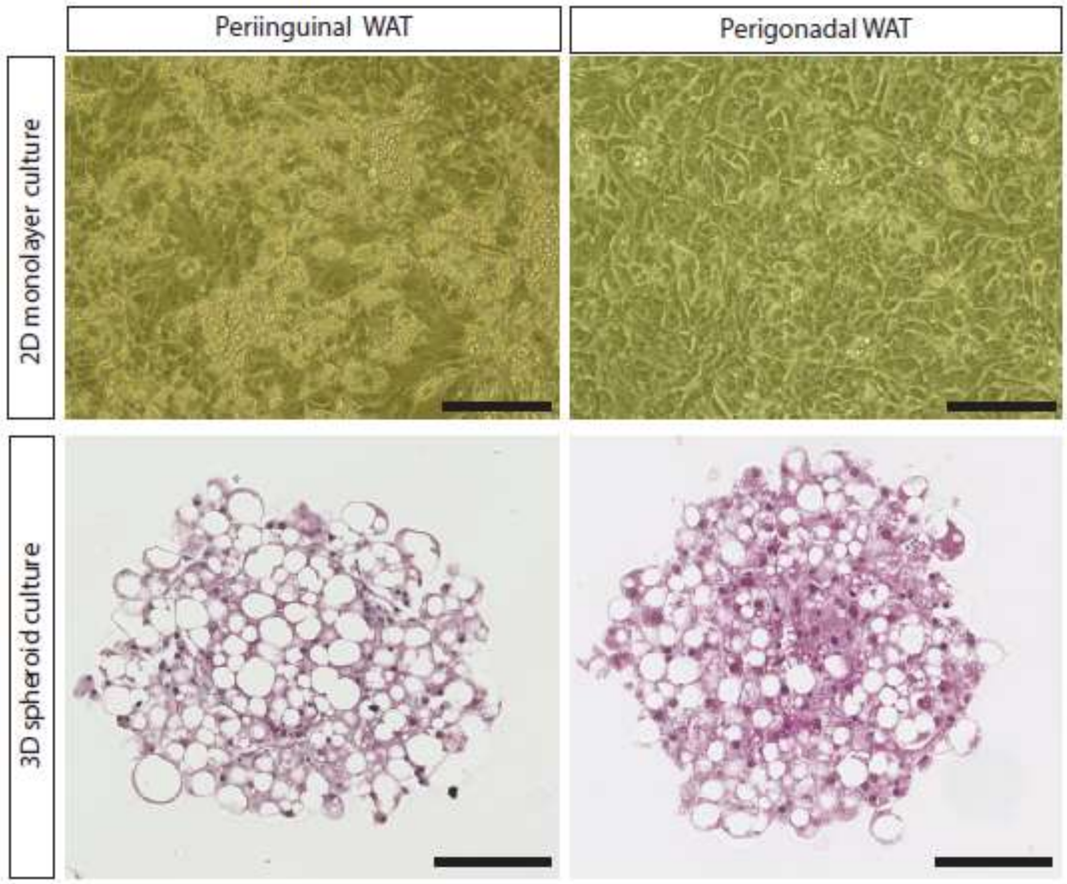

Supplementary Figure 4: Differentiation of inguinal and perigonadal in 2D culture. Note that while a considerable fraction of inguinal subcutaneous adipocytes differentiate into multilocular cells with small lipid droplets in 2D culture, differentiation and lipid accumulation cannot be observed in 2D cultures from perigonadal preadipocytes. In contrast, preadipocytes from both depots differentiate well in 3D culture. Scale bars = 100µm.

Supplementary Table 1:Probes and primer sequences used for gene expression analysis.

Gene	Species	Taqman Probe ID	SYBR Green Primer Sequences
ADIPOQ	Human	hs00605917_m1	-
B2M	Human		for: AAGGACTGGTCTTTCTATCTC<br>rev: GATCCCACTTAACTATCTTGG
FABP4	Human	hs01086177_m1	-
PLIN4	Human	hs00287411_m1	-
PPARG	Human	hs00234592_m1	-
Adipoq	Mouse	-	for: CAACCAACAGAATCATTATGA<br>rev: ACACCGTGATGTGGTAAGAGA
Elovl6	Mouse	-	for: TGCAGGAAAACTGGAAGAAGTCT<br>rev: ATGCCGACCACCAAAGATAAA
Fabp4	Mouse	-	for: TTCGATGAAATCACCGCAGA<br>rev: GGTCGACTTTCCATCCCACTT
Hprt	Mouse	-	for: TGGCCATCTGCCTAGTAAAGC<br>rev: GGACGCAGCAACTGACATTTC
Lep	Mouse	-	for: AAGACCATTGTCACCAGGATCAA<br>rev: ACCCTCTGCTTGGCGGATA
Lipe	Mouse	-	for: ACTTCTGGAAAGCCTTCTGGAACA<br>rev: GATGCCATGTTGGCCAGAGAC
Plin1	Mouse	-	for: AACAGCATCAGTGTGCCCATT<br>rev: CCATCCCCAAGGCAAGCT
Plin4	Mouse	Mm00491061_m1	-
Pparg2	Mouse	-	for: AGTGTGAATTACAGCAAATCT<br>rev: CGAGTGGTCTTCCATCACGG
Scd1	Mouse	Mm01197142_m1	-
GFP	N/A	-	for: AGCAAAGACCCCAACGAGAAG<br>rev: GTCCATGCCGAGAGTGATCC
